# Functional analysis of ultra high information rates conveyed by rat vibrissal primary afferents

**DOI:** 10.3389/fncir.2013.00190

**Published:** 2013-12-05

**Authors:** André M. Chagas, Lucas Theis, Biswa Sengupta, Maik C. Stüttgen, Matthias Bethge, Cornelius Schwarz

**Affiliations:** ^1^Systems Neurophysiology Group, Werner Reichardt Center for Integrative Neuroscience, University TübingenTübingen, Germany; ^2^Department for Cognitive Neurology, Hertie Institute for Clinical Brain Research, University of TübingenTübingen, Germany; ^3^Computational Neuroscience Group, Werner Reichardt Center for Integrative Neuroscience, University TübingenTübingen, Germany; ^4^Graduate School for Neural and Behavioural Sciences, University TübingenTübingen, Germany; ^5^Wellcome Trust Centre for Neuroimaging, University College LondonLondon, UK; ^6^Centre for Neuroscience, Indian Institute of ScienceBangalore, India; ^7^Department of Neuroscience, Erasmus Medical CenterRotterdam, Netherlands; ^8^Department of Biopsychology, University of BochumBochum, Germany; ^9^Max Planck Institute for Biological CyberneticsTübingen, Germany; ^10^Bernstein Center for Computational Neuroscience, University of TübingenTübingen, Germany

**Keywords:** rat, whisker, vibrissae, primary afferents, tactile coding, information theory, spike-triggered mixture model

## Abstract

Sensory receptors determine the type and the quantity of information available for perception. Here, we quantified and characterized the information transferred by primary afferents in the rat whisker system using neural system identification. Quantification of “how much” information is conveyed by primary afferents, using the direct method (DM), a classical information theoretic tool, revealed that primary afferents transfer huge amounts of information (up to 529 bits/s). Information theoretic analysis of instantaneous spike-triggered kinematic stimulus features was used to gain functional insight on “what” is coded by primary afferents. Amongst the kinematic variables tested—position, velocity, and acceleration—primary afferent spikes encoded velocity best. The other two variables contributed to information transfer, but only if combined with velocity. We further revealed three additional characteristics that play a role in information transfer by primary afferents. Firstly, primary afferent spikes show preference for well separated multiple stimuli (i.e., well separated sets of combinations of the three instantaneous kinematic variables). Secondly, neurons are sensitive to short strips of the stimulus trajectory (up to 10 ms pre-spike time), and thirdly, they show spike patterns (precise doublet and triplet spiking). In order to deal with these complexities, we used a flexible probabilistic neuron model fitting mixtures of Gaussians to the spike triggered stimulus distributions, which quantitatively captured the contribution of the mentioned features and allowed us to achieve a full functional analysis of the total information rate indicated by the DM. We found that instantaneous position, velocity, and acceleration explained about 50% of the total information rate. Adding a 10 ms pre-spike interval of stimulus trajectory achieved 80–90%. The final 10–20% were found to be due to non-linear coding by spike bursts.

## Introduction

Primary afferents of the rodent whisker-related tactile system are classically categorized by their response pattern to ramp-and-hold stimuli (Gibson and Welker, 1983a,b). One class of neurons responds to a ramp-and-hold stimulus with a phasic onset burst, followed by sustained firing during the hold phase of the stimulus [dubbed “slowly adapting” (SA)]; the other class responds only during the ramp phase and is therefore called “rapidly adapting” (RA). The picture that emerges from these and other studies is that SA spiking encodes stimulus velocity and position during the ramp and the hold phase of the stimulus, respectively, while RA spiking encodes solely velocity during the ramp phase. Moreover, dynamic velocity ranges for SA and RA afferents seem to be complementary, with SA spiking best representing low velocities (<750°/s) and RA spiking best representing high velocities (>750°/s; Shoykhet et al., 2000; Stüttgen et al., 2006). While this notion is supported by studies using step-like stimuli (Stüttgen et al., 2006, 2008) it does not seem to apply in case of more complex stimuli (Jones et al., 2004a,b): Low-pass filtered white-noise stimuli activate both classes of primary afferents in an utmost precise way. Spike-triggered averages [or the related filter kernels that transform stimuli into spike trains, (Bialek et al., 1991)] computed from primary afferent responses to white-noise stimuli exhibit shapes that in most cases could not be described as uniquely encoding either position, velocity, or acceleration (Jones et al., 2004a). Furthermore, spike-triggered averages differ according to the frequency range of the white-noise stimuli, suggesting more complex response properties than captured by analyses using step-like stimulation.

In this study we use time-varying stimuli and information theoretical analyses to assess how much information about the whisker trajectory is conveyed by the responses of RA and SA cells. We first use the “direct method” (DM) (De Ruyter Van Steveninck et al., 1997) for estimating how much information about the stimulus is transmitted by these neurons. However, while this method is popular due to its simplicity and generality, it does not provide a functional description of the neural response properties. A widespread method for describing the function of a neuron is to estimate filters, for example based on linear-non-linear cascade models, which has also been applied to the vibrissal system (Petersen et al., 2008; Estebanez et al., 2012). However, primary afferents turn out to be sensitive to frequencies that are beyond the cut-off frequency of the test stimuli used due to mechanical constraints. Since this impedes the interpretability of the filter shapes (Appendix 1), we opted for an alternative approach: Information theoretic methods were employed to quantitatively compare information content of spike responses obtained from a novel generative model using fits of multiple Gaussians (STM, Theis et al., 2013). With this technique we obtain similar information rates as for the DM, but are able to dissect the transduced information on a mechanistic level. Importantly, the encoding in this model captures multiple preferred stimuli (i.e., multiple specific combinations of kinematic parameters) and dependencies on spike history introduced by bursting. The total information flow in primary afferents that is explained by this model is amongst the highest ever reported in sensory afferents or receptors.

## Materials and methods

### Animals

Four female adult (12–18 weeks) Sprague-Dawley rats weighing 240–300 g were used in this study. All experimental and surgical procedures were carried out in accordance with the policy on the use of animals in neuroscience research of the Society for Neuroscience and German law.

### Surgery and recording

Anesthesia was introduced with a combination of ketamine and xylazine (100 and 10 mg/kg body weight, respectively) injected intraperitoneally, and maintained with 1–2% isoflurane (1-Chloro-2,2,2-trifluoroethyl-difluoromethylether) in medical oxygen. The concentration of isoflurane was chosen such that the animal's pain reflexes, tested by a pinch of the hind paw with a forceps, remained absent throughout the entire experiment. The animal's temperature was monitored by a rectal probe and kept constant at 37°C by a heating pad (Harvard apparatus homoeothermic blanket control unit). At the end of the experiment, the rat was killed with an overdose of pentobarbital.

After induction of the anesthesia, the animal was head-fixed in a stereotaxic frame (Kopf Instruments, Tujunga, USA) and a craniotomy was performed to expose the brain's right hemisphere, which was then partially aspirated to reveal the dura mater covering the trigeminal ganglion (TG). After careful hemostasis, the dura overlying the ganglion was teased away, and laboratory-built pulled and ground glass-coated platinum tungsten electrodes (80 μm shank diameter; 23 μm diameter of the metal core; free tip length ~8 μm; impedance, 3–6 MΩ; Thomas Recording, Giessen, Germany) were lowered into the ganglion until units responding to manual whisker stimulation were encountered. Bandpass filtered (300–10,000 Hz) voltage traces were digitized at a 20 kHz sampling rate using an extracellular amplifier (MultiChannel Systems, Reutlingen, Germany).

**Figure 1:**
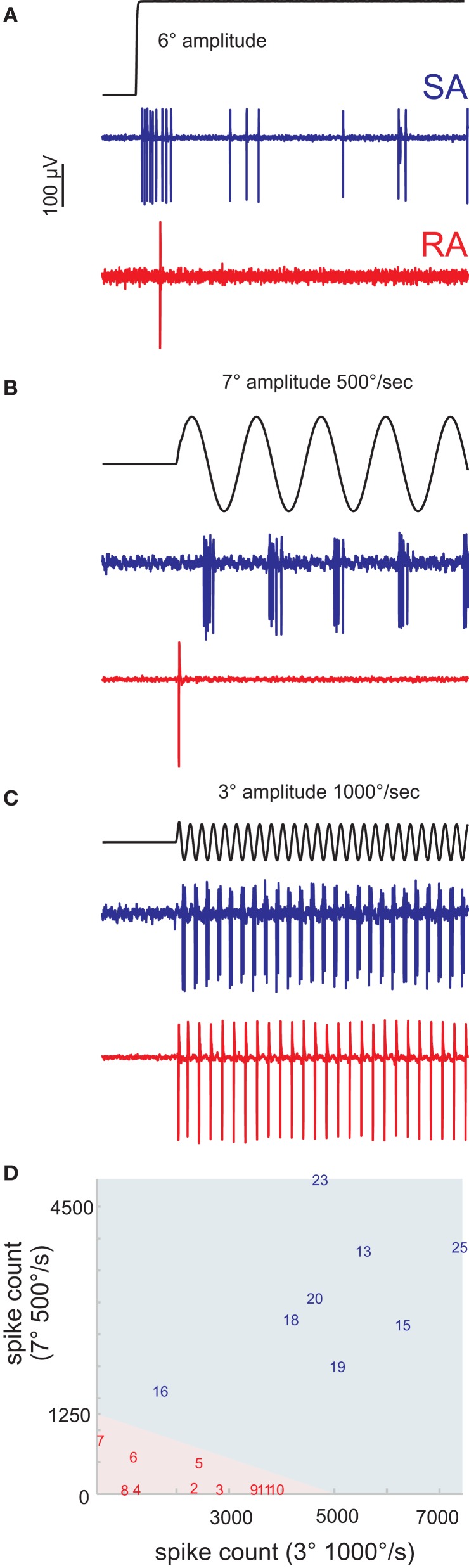
**Classification of primary afferents. (A)** Typical responses of rapidly (RA) and slowly adapting (SA) primary afferents to a ramp-and-hold stimulus in the preferred direction of the cell. **(B,C)** Discriminative responses of RA and SA units to sine waves of different amplitude and peak frequency. **(B)** Slow, high amplitude sine waves are not responded by RAs but by SAs. **(C)** Fast, low amplitude sine waves evoke responses in both cell classes. **(D)** A number of preliminary recordings were used to delineate the responses of RA and SA cells to the two sine wave stimuli (light red and blue background). The units reported in this study are labeled by a number. The numbering of cells and coloring (red/blue) according to cell class is consistent throughout all figures to allow the cross referencing of individual cells.

Units were isolated using electrode step movements of 1 μm amplitude. The present sample consists of unequivocal single units—the waveforms exceeded the noise amplitude by factors of 3–18 (median 5) (see example traces in Figures 1A–C, 6A). The whisker whose deflection drove the responses was identified by a hand held probe, and deflected in different directions to find out the direction that gave the maximal response. The whisker was then placed inside a polyimide tube attached to an actuator. The actuator consisted of a galvanometer motor (model 6220H, Cambridge Technology, Lexington, USA) controlled by a closed-loop system (micromax 67145 board, Cambridge Technology). The command voltage (±10 V) was provided by custom code programmed in LabView® 2009 (National Instruments, Austin, USA). The galvanometer arm was aligned such that stimulation occurred along the axis of the cell's preferred direction, starting at the whisker's resting position. The end of the polyimide tube was at a distance of 5 mm from the rat's skin.

### Cell classification

An initial number of single units (*n* = 18) was recorded to establish an efficient way to classify primary afferents into SA and RA cells. The idea of these preliminary experiments was to use the well-established ramp-and-hold stimuli as a reference to calibrate a classifier based on the presentation of just two sinusoidal stimuli that later can be applied efficiently in the experiments involving time-consuming white noise stimulations. The preferred deflection direction of the neuron under study was identified using a hand-held probe. Thereafter, these receptive fields of the units were carefully mapped using the galvanometer motor. Twenty-five ramp-and-hold stimuli with amplitudes of 1, 2, 4, 8, and 12°, and peak velocities of 62, 250, 500, 1000, 1500°/s were applied in the unit's preferred direction (stimulus waveforms were identical with the ones used in Stüttgen et al., 2006) (Figure 1A). In addition, we applied two sine wave stimuli, the first (Figure 1B) with an amplitude of 7° and a peak velocity of 500°/s, and the second (Figure 1C) with 3° and 1000°/s, respectively. These sine waves should be optimally tuned to tap into the two perceptual channels carried by SAs and RAs (Stüttgen et al., 2006), i.e., separate SA from RA by silencing one while efficiently stimulating the other. However, our stimulator could not be tuned to deliver stimuli in both ranges without severe deterioration of some trajectories due to manipulator resonances. The two stimuli are therefore a compromise, but they can be safely delivered with one and the same stimulator (the galvanometer) and were found to effectively separate the two groups. Figure 1D depicts the linear classifier (indicated by the background color) estimated using these preliminary recordings and stimulation with step stimuli. The responses of all cells to the sine waves in the final data set (8 SA and 10 RA) were classified accordingly (indicated by numbers).

### White noise stimuli

**Figure 2:**
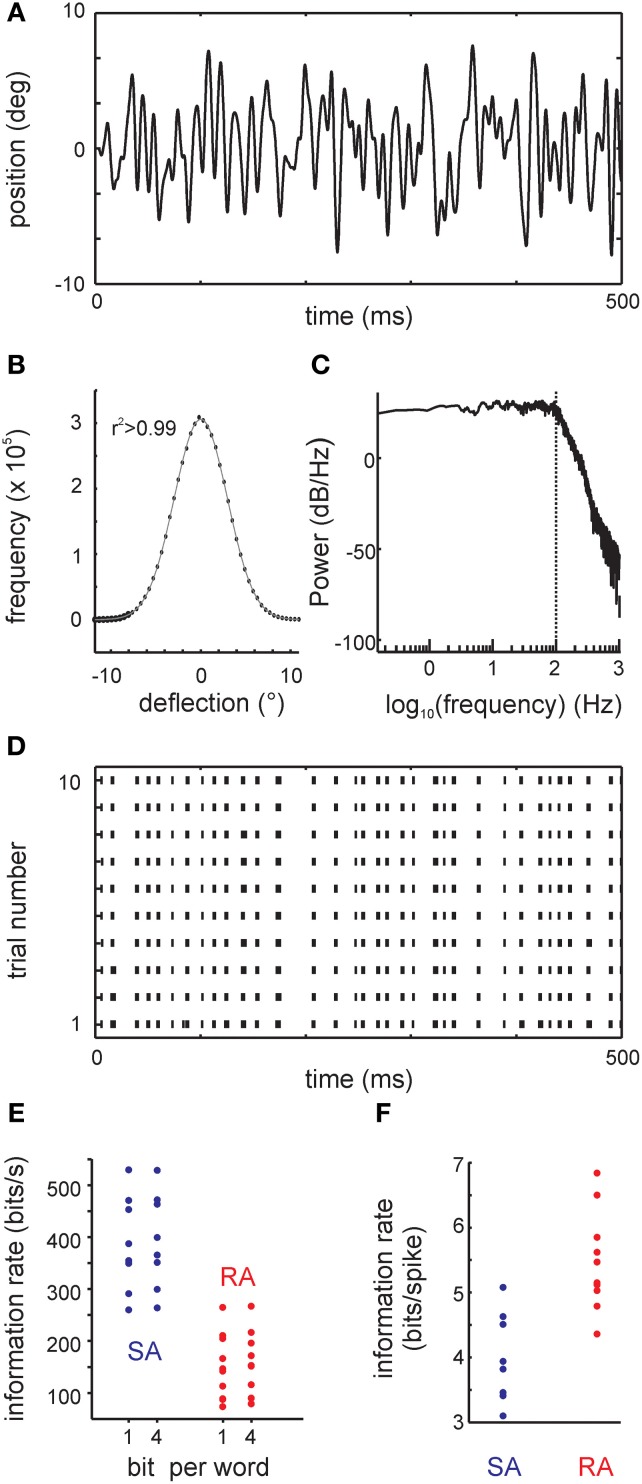
**The filtered white noise stimulus and results obtained with a classical information theoretical analysis: the direct method (DM). (A)** Example trace of the whisker's position. **(B)** The distribution of positions (circles) and the fit to a Gaussian (line). The coefficient of determination of the fit was *r*^2^ > 0.99. **(C)** The stimulus power spectrum showing a flat density below 100 Hz (vertical dotted line). Above 100 Hz, there is a smooth roll-off. **(D)** Responses to the filtered white noise stimulus are very precise as demonstrated by the raster plot of one example primary afferent [response to the trace shown in **(A)**]. **(E)** Information rate (bit/s) calculated using DM for all cells in the sample. Information rate with bin width of 1 ms using 1 bit words (DM1.1) and 4 bit words (DM1.4) are shown. **(F)** Information rate converted into units of bits per spike (DM1.1).

White noise stimuli were generated and filtered online (Butterworth filter of order 5, sampling rate 20 kHz) with LabVIEW 2009® (National Instruments). The movement trajectories as assessed by the galvanometer output (Figure 2A), and measurements using photodiodes (Stüttgen et al., 2006) were identical. The stimulus was filtered Gaussian white noise with a flat spectrum up to an edge frequency of 100 Hz; the Gaussian fit of the amplitude distribution was of excellent goodness (*r*^2^ = 0.9999) (Figures 2B,C). At each time bin (50 μs), the sample of the stimulus consisted of a triplet containing position, velocity, and acceleration. The 3D ellipsoid delimiting the 3 standard deviation limit of the stimulus distribution (containing 99.7% of the data) intercepted the axes at ±10° amplitude, ± 4 × 10^3^°/s velocity, and ± 2 × 10^6^°/s^2^ acceleration. The spike response of primary afferents to 50 trials of band-pass filtered Gaussian white noise was recorded. One trial consisted of two epochs of filtered white noise stimulation (epoch duration 5 s), separated by a 2 s inter-stimulus interval. The two epochs consisted of “frozen noise” (repeated presentation of the same stimulus) and “unfrozen noise” (a different stimulus on every trial). All cells in the sample were recorded using the stimulus characterized above. Some were recorded additionally with a second stimulus with identical characteristics except that it was attenuated to about half amplitude (3 standard deviations of ±5.1°). In this report we generally describe the results obtained with the larger amplitude stimulus. The smaller one was used as a control to assess whether the estimated mutual information is dependent on stimulus amplitude (cf. Figure 10D).

### Classical information theoretical analysis

Only unequivocal single unit data entered the present data set. All spike wave amplitudes were far above triple noise level and could be easily isolated by applying a threshold to the voltage trace (examples in Figures 1, 6A). The information rates transmitted by the different neurons were calculated using the “DM” (De Ruyter Van Steveninck et al., 1997), which estimates information transfer directly from the spike trains. DM is commonly thought to offer a strategy to measure mutual information that is free from assumptions regarding the encoding of the stimulus (De Ruyter Van Steveninck et al., 1997; Borst and Theunissen, 1999). It involves an estimate of the marginal (or total) entropy of the spike trains, *H*[*s*], and an estimate of the conditional (or noise) entropy of the spike trains conditional on the stimulus, *H*[*s*|*x*]. Mutual information between spike trains and stimuli is defined as the difference of these two entropies, i.e.,

(1) I​[s,x]=H​[s]−H[s|x].

The marginal entropy is estimated by building a histogram of binary words of fixed length randomly selected from the spike trains, *w* ∈ {0, 1}^*N*^. Using *N* bit words, a histogram over 2^*N*^ possible states is obtained. Its entropy is used as an estimator of the marginal entropy,

(2) $$H\text{​}\left[s\right]=-\sum_{w}p(w)\log p(w).$$

For estimating the conditional entropy, one such histogram is computed for each point in time from repeated trials with a fixed (frozen) stimulus. Afterwards, the entropies of all histograms are averaged,

(3) $$H[s|x]=-\frac{1}{T}\sum_{t}\sum_{w}p_{t}(w)\log p_{t}(w).$$

We obtained both estimates from 50 trials with frozen stimuli. For the word length and bin size we used 1 bit and 1 ms, respectively. Dividing the result by the word length and multiplying by the sampling rate yields an estimate of the mutual information per time interval, or information rate.

### Spike-triggered stimulus ensembles

**Figure 3:**
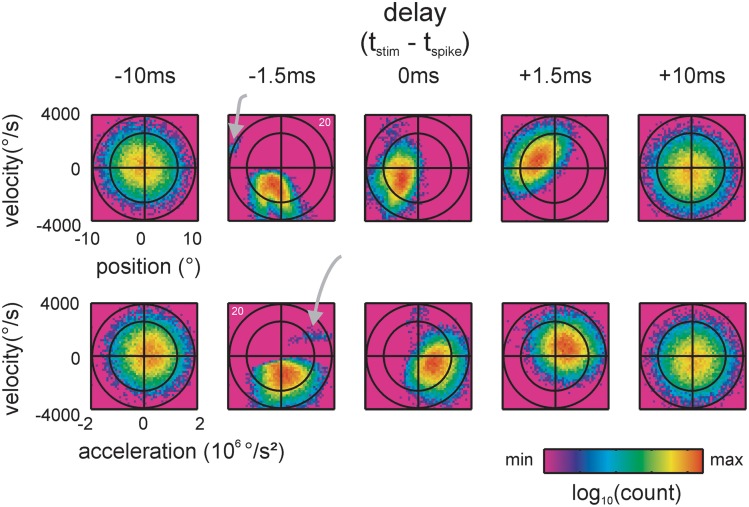
**Spike-triggered kinematic features**. Two dimensional projections, taken from the 3D distributions of stimulus space spanned by position, velocity and acceleration, are shown. Top: position and velocity. Bottom: acceleration and velocity. The two ellipses (black lines, scales are chosen such that they appear as circles) indicate the total stimulus space (2 times and 3 times standard deviation of the 2D Gaussian). 2D histograms of the spike-triggered stimulus ensembles (color-coded) are plotted on top. Five delays between the occurrence of the spike and the stimulus feature are shown. At negative delays the spike follows the occurrence of the stimulus feature. Note the sharp and lobed sub-space (a separated preferred stimulus/lobe is marked by gray arrows) emerging when the stimulus is sampled 1.5 ms before the spike. (data of cell 20, cf. Figure 2D).

**Figure 4:**
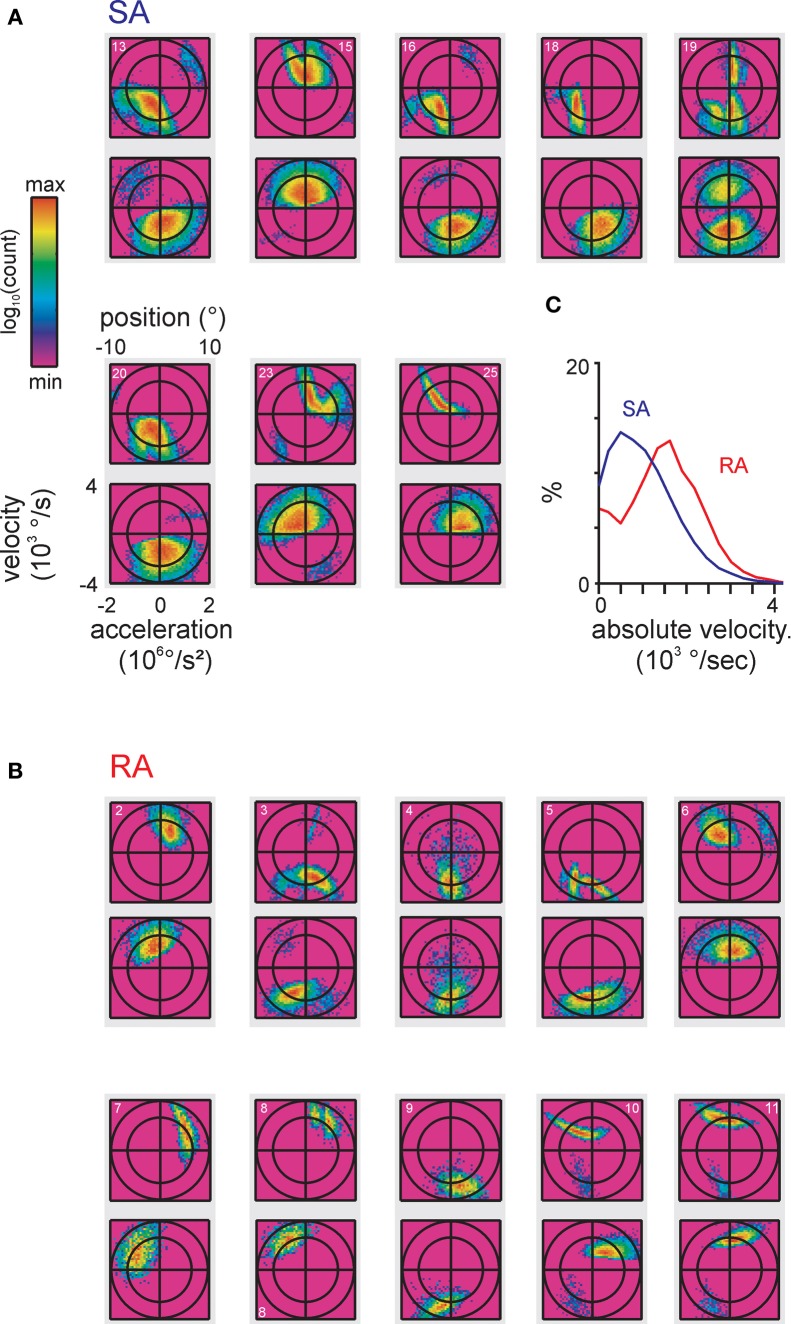
**Encoded instantaneous stimulus features for our total sample of primary afferents. (A,B)** original 3D stimulus space (as done in Figure 3). The spike-triggered stimulus ensembles found by maximizing the Kullback-Leibler Divergence (KLD) between stimulus distribution and spike-triggered stimulus ensemble are shown. Axis scaling and labeling is identical on all plots and is only shown once for clarity. The data pertaining to each cell are plotted onto a gray rectangle, the top plot depicts position vs. velocity, the bottom plot depicts acceleration vs. velocity. The cell numbers relating to Figure 2D are given in the top plots. **(C)** Average of one-dimensional projections onto the velocity axis showing different spike-triggered distributions of velocities in SA vs. RA cells distributions were sampled at each cell's optimal delay (cf. Figure 3).

As the estimated temporal integration window of the primary afferents appeared to be very small, we first built an encoding model that assumes near-instantaneous encoding. To this end, we used spike-triggered distributions of position, velocity, and acceleration at a single fixed delay. For visualization purposes, we decompose these three-dimensional distributions into two two-dimensional distributions (Figures 3, 4A,B). Since our stimulus is a Gaussian process and differentiation is a linear operation, the resulting position, velocity, and acceleration at any point in time are also Gaussian distributed. We indicate 2 and 3 standard deviations of these by ellipses (Figures 3, 4A,B), which contain ~95.5 and ~99.7% of all possible kinematic values in the stimulus, respectively. The spike-triggered stimulus ensembles are superimposed onto the prior stimulus distributions as color-coded histograms. We assessed the optimal delay by determining the maximum information that can be gained about the stimulus from observing a spike or no spike in a single time bin, varying the delays between −10 and +10 ms in steps of 50 μs. This information is measured in bits by the Kullback-Leibler divergence (KLD):

(4) $$D\text{​}\left(p\left(x|s=1\right)\|p(x)\right)=\sum_{x}p(x|s=1)\log_{2}\frac{p(x|s=1)}{p(x)}$$

where *D* is the KLD, *x* the instantaneous stimulus at one delay, and *s* is a binary value signifying the presence/absence of a spike.

### Spike train autocorrelograms corrected for stimulus correlation

To extract significant spike patterns (doublets and triplets of spikes) from single unit recordings, we used autocorrelogram (AC) analysis. A feature in the AC of a spike train in our sample could be due to intrinsic spike patterns, but alternatively may be trivially explained by stimulus correlation (the stimulus contains correlations within a window of ca. 10 ms due to low-pass filtering). To correct for this latter unwanted feature, the ACs were converted to the frequency domain yielding the power spectra of the spike train and stimulus time series (Wiener Khinchin theorem). Then the bin-by-bin ratio of stimulus spike train power spectra was calculated and transformed back to the time domain, resulting in *corrected* ACs in which the autocorrelation of spike trains that was due to stimulus correlation was eliminated. Significant bursting was identified by peaks in the corrected AC that surpassed a prediction interval (PI), which was assessed from the 5 to 95% percentile of a bootstrapped distribution (1000x) of corrected ACs obtained using randomly shuffled spike times.

### Generative encoding model using a fit of multiple gaussians

We modeled the spike-triggered and non-spike-triggered stimulus ensembles using mixtures of Gaussians [spike-triggered mixture model, STM, as detailed in Theis et al. (2013)]. An application of Bayes' rule allows us to turn these distributions into a probabilistic model of the neuron's behavior. If *x*_*t*_ is the stimulus at time *t* and *s*_*t*_ ∈ {0, 1} indicates the absence or presence of a spike at time *t*, then the probability of observing a spike is given by

(5) $$p\text{​}\left(s_{t}=1|x_{t}\right)=\frac{p\text{​}\left(x_{t}|s_{t}=1\right)\text{​}p\text{​}\left(s_{t}=1\right)}{p\text{​}\left(x_{t}|s_{t}=1\right)p\text{​}\left(s_{t}=1\right)+p\text{​}\left(x_{t}|s_{t}=0\right)\left(1-p\text{​}\left(s_{t}=1\right)\right)}$$

where *p*(*x*_*t*_|*s*_*t*_ = 1) is the spike-triggered distribution and *p*(*x*_*t*_|*s*_*t*_ = 0) is the distribution of stimuli when there is no spike. We represent both distributions as mixtures of Gaussians. *p*(*s*_*t*_ = 1) is simply the average number of spikes per bin.

This model can be extended in a principled manner to include dependencies on other factors such as the spike history. To obtain a potentially more powerful model, we included stimulus windows of up to 20 ms and a variable τ_*t*_ for the time past since the last spike. Thus, *p*(τ_*t*_|*s*_*t*_ = 1) is the inter-spike interval distribution. We made the simplifying assumption that the stimulus and τ_*t*_ are conditionally independent, that is *p*(*x*_*t*_, τ_*t*_|*s*_*t*_) = *p*(*x*_*t*_|*s*_*t*_)*p*(τ_*t*_|*s*_*t*_). This is also known as the naïve Bayes assumption and is often employed in classification. It is a common heuristic which can lead to good results even when the naïve Bayes assumption is not satisfied (Zhang, 2004; Bishop, 2006). Taken together, our model is characterized by three factors:

(6) $$p(s_{t}|x_{t},\tau_{t})\propto p(x_{t}|s_{t})p(\tau_{t}|s_{t})p(s_{t})$$

When *s*_*t*_ = 1, the three factors on the right-hand side of the equation correspond to the spike-triggered distribution, the inter-spike interval distribution, and the prior probability of observing a spike, respectively. Here we used inter-spike interval histograms for estimating the conditional distributions *p*(τ_*t*_|*s*_*t*_ = 1) and *p*(τ_*t*_|*s*_*t*_ = 0).

An alternative view of the model is expressed by

(7) $$p\text{​}\left(s_{t}=1|x_{t},\tau_{t}\right)=\frac{1}{1+\text{exp}\left(-f(x_{t},\tau_{t})\right)}$$

where

(8) $$\begin{array}{l} f\text{​}\left(x_{t},\tau_{t}\right)=\log p\text{​}\left(s_{t}=1\right)+\log p(x_{t}|s_{t}=1)+\log p(\tau_{t}|s_{t}=1)\\ \;-\log p(s_{t}=0)-\;\log p(x_{t}|s_{t}=0)-\log p(\tau_{t}|s_{t}=0). \end{array}$$

As can be seen from Equations 7, 8, the firing rate of the model is determined through a linear integration of a set of non-linear features and an application of a sigmoid logistic function (*cf.* Figure 9).

For the DM, estimation of the information transmitted by each neuron requires us to estimate two entropies: a marginal entropy *H*[*s*] and a conditional entropy *H*[*s*|*x*] of spike trains *s*. The latter can be decomposed as follows:

(9) $$H\text{​}\left[s|x\right]=-E[\log p(s|x)]=-\sum_{t=1}^{T}E[\log p(s_{t}|x,s_{<t})],$$

where *s*_<*t*_ is the history of spikes before time *t*. If we assume that *s*_*t*_ does not depend on the entire stimulus but only on a short stimulus window *x*_*t*_ preceding it, and we further assume that *s*_*t*_ depends only on the position of the most recent spike, then *p*(*s*_*t*_|*x, s*_<*t*_) can be replaced by (*s*_*t*_|*x*_*t*_, τ_*t*_). After training our model to approximate *p*(*s*_*t*_|*x*_*t*_, τ_*t*_) on the unfrozen trials, we estimated entropies by averaging −log *p*(*s*_*t*_|*x, s*_<*t*_) over the frozen trials. *H*[*s*] was estimated in the same manner but using a model for *p*(*s*_*t*_|τ_*t*_) ∝ *p*(τ_*t*_|*s*_*t*_)*p*(*s*_*t*_), that is, ignoring the stimulus.

Using stimulus windows of up to 10 ms before the spike led to an improved predictive performance of our model and an increase in estimated information rate. For 10 ms windows, the dimensionality of the stimulus was first reduced to 10 using PCA. The parameters of the model were initialized by the fits of mixtures of Gaussians to the spike-triggered and non-spike-triggered stimulus ensembles and the inter-spike interval histograms. As a fine-tuning step, the expected conditional log-likelihood of the model, *E*[log *p*(*s*_*t*_|*x*_*t*_, τ_*t*_)], was directly optimized using the quasi-Newton method BFGS (Nocedal and Wright, 1999). This last step, while computationally more expensive, was necessary to obtain optimal performance.

## Results

We recorded spikes from 18 single primary afferents in the TG of anesthetized rats. All recorded primary afferents were responsive to only one whisker. After we determined their preferred direction of deflection, two sinusoidal whisker deflections were applied along the axis of preferred direction to classify units into SA and RA (see materials and methods, Figure 1). One recording contained 50 trials, each of which consisted of two 5-s epochs of stimulation with low-pass filtered Gaussian white noise (cut-off frequency 100 Hz; Figures 2A–C), also presented along the axis of preferred direction. These epochs were separated by 2 s of silence and consisted of (1 frozen and 2) unfrozen white noise. The present data base contains 8 SA and 10 RA which were confronted with the full stimulus set.

### Information rates (“direct method”)

Primary afferents responded to low-pass filtered white noise stimulation (Figures 2A–C) with very precise and repeatable spike trains (Figure 2D). As a reference measure, we first estimated the mutual information between stimulus and spike trains using the classical “DM” with 1ms bin size and 1-bit word estimates (DM1.1) (Figures 2E,F). The median information rate was 371.07 bit/s (3.88 bit/spike) for SAs (maximum 529.47 bit/s, 5.08 bit/spike), while it reached a median of 144.28 bit/s (5.31 bit/spike) for RAs (maximum 264.81 bit/s, 6.84 bit/spike). The maximum information rates in vibrissa-related primary afferents were thus in the range of the highest ones that have been reported with similar methods in other model systems (Borst and Theunissen, 1999).

The information rate estimates are dependent on the bin size parameter, and would come out even larger for smaller bin sizes. Following common practice, we used a bin size of 1 ms, which can be justified by the width of an action potential. For the sake of reliability, all information rates reported are based on 1-bit word estimates. Using 4-bit words instead only had a small effect on the estimated information rates of all primary afferents (Figure 2E).

### Spike- and pattern-triggered stimulus ensembles

Traditionally, position, velocity, and acceleration are used to describe the response properties of RA and SA cells. Figure 3 exemplifies spike-triggered stimulus ensembles consisting of triples of kinematic values—instantaneous position, velocity and acceleration, measured at different delays with respect to the spike times. The term “instantaneous” as we use it here means that the values of kinematic variables were taken from a single 50 μs bin (the resolution of stimulus presentation) preceding the spike by a certain time interval. For visualization purposes, the spike-triggered stimulus ensembles in Figure 3 are split into two 2D projections spanned by either position and velocity or velocity and acceleration. The stimulus ensembles, depicted as color-coded histograms, were obtained for varying time offsets to the spike. The spike-triggered stimulus ensemble extracted 10 ms before the spikes (i.e., with a delay of −10 ms) occupied a large portion of the total stimulus space (leftmost column). Some 8.5 ms later, at a delay of −1.5 ms, a sharply structured sub-space emerged that occupied the smallest space encountered with all delays. At this delay, the spike-triggered ensemble takes the shape of a complex set of stimulus features—the sub-field can be described as a composition of two partially confluent lobes plus one lobe clearly separated from the other two (gray arrow). At delays of 0 and +1.5 ms, the structure of the sub-space blurred and spread out to cover again a large portion of the total stimulus space 10 ms after the spike (delay +10 ms) (Figure 3). Using the Kullback-Leibler divergence (KLD, see materials and methods) between the spike-triggered and total stimulus ensembles (characterized by the three-dimensional distribution of spike-triggered position, velocity, and acceleration), we assessed the optimal delay by determining the maximum information that can be gained about the stimulus from observing a spike or no spike in a single time bin varying the delays between −10 and +10 ms in steps of 50 μs. Optimal delays were not significantly different for SA vs. RA and ranged around 1.5 ms (SA: median −1.45 ms (range −2.4, −0.8); RA: −1.4 ms (range −3.45, −0.45); Mann–Whitney *u* test, *p* = 0.85). This is consistent with the view that these delays are solely due to stimulus transduction in the follicle and spike conduction to the somatic recording site in the ganglion.

The spike-triggered ensembles of all cells in the data set observed with these optimal delays are depicted in Figures 4A,B. In general, the lobes were of ellipsoid form with the longer axis often pointing at oblique directions, i.e., not compatible with tuning to just one kinematic parameter. Moreover, whenever multiple lobes were present, typically their orientation in stimulus space differed. There was no qualitative difference in the complexity and size of subspaces covered by SA and RA cells. Both primary afferent classes showed clear features when plotting velocity together with acceleration, albeit in SA cells the distributions along the acceleration axis were broader, typically covering a substantial part of possible stimulus accelerations. Such complex features were not expected based on knowledge gained from ramp-and-hold stimuli to which primary afferents show monotonically increasing tuning curves (Stüttgen et al., 2006). However, a more general observation of the cited study, namely that SA afferents encode a lower velocity range than RA afferents, was confirmed by the present responses to complex stimuli as revealed by comparing the distribution of the spike-triggered stimulus ensemble along the velocity axis pooled over all RA and SA cells, respectively (Figure 4C).

**Figure 5:**
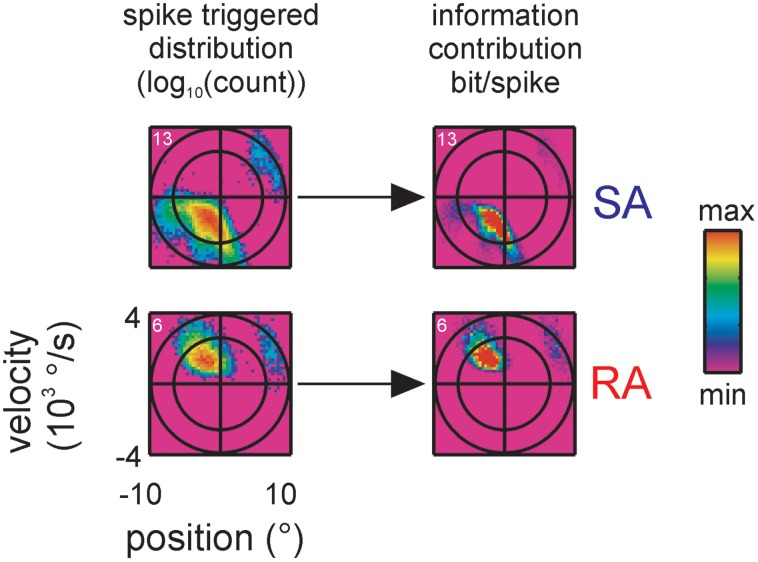
**Information contribution of extra lobes**. Encoded stimulus features of two representative primary afferents containing multiple lobes, one SA (top, cell #13) and one RA (bottom, cell #6), are shown (conventions for axes and ellipsoid as in Figure 3). On the left, the spike triggered stimulus distributions are re-plotted from Figure 4 for comparison. On the right the information contribution of each stimulus bin is plotted (term within the sum operator in Equation 4). Information contribution of the extra lobe scales with the spike triggered distribution indicating that the sensitivity of the extra lobe does not grossly deviate from the one of the main lobe.

In view of the complex, lobed spike triggered stimulus distributions we asked the question how much information the extra lobes contribute to the total information rate. For instance it might be possible that information rate originates largely from extra lobes because these stimulus ranges exert an increased influence on the cell's spiking. To this end we derived the contribution of each stimulus triple to the total information by computing the term $p(x|s=1){\text{log}}_{2}\frac{p(x|s=1)}{p(x)}$ (as shown by Equation 4 the sum of this term over all bins yields the KLD). Figure 5 plots this measure (across position and velocity) next to the spike triggered distributions for two representative neurons (one SA, one RA; all cells in the sample with multiple lobes show similar results). By comparing these plots with the spike-triggered distributions, it can be seen that the lobe's contributions to information transfer is similar to the probability to trigger a spike. This indicates that the amount of information transferred per spike is largely the same across the responsive stimulus sub-space.

### Spike patterns and multiple preferred stimuli

**Figure 6:**
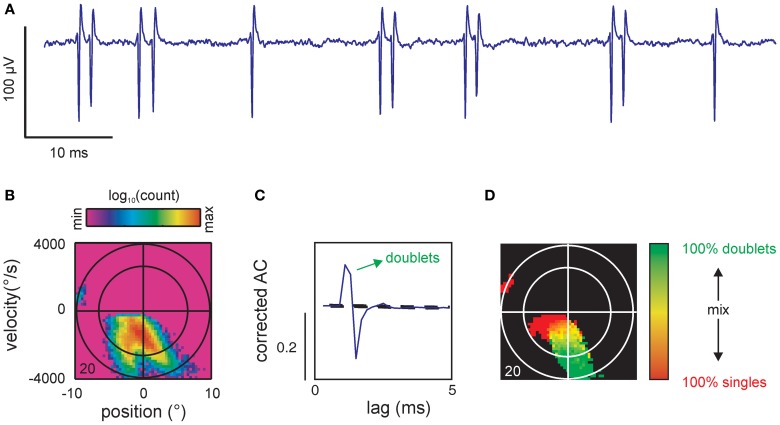
**Spike patterns. (A)** Voltage trace of single unit recording showing 2 single spikes and 5 doublets. **(B)** Spike-triggered stimulus ensemble of cell 20. **(C)** Corrected autocorrelogram (AC) showing a prominent peak far exceeding the prediction interval [PI, (5, 95%), broken lines]. The peak is followed by a trough departing below the PI likely indicating a refractory period after the second spike of the doublet. The ordinate scales correlation coefficient (*r*). Same cell as **(B)**. **(D)** Coding of single spikes vs. doublets. Doublets were identified by searching the spike train for spike intervals corresponding to the interval as specified by the significant peak in the AC [cf. **(C)**]. To eliminate spurious spiking the map only contained color in bins that exceeded a total count of 5 events (spikes or doublets). Conventions for axes and ellipsoid in spike triggered ensembles as in Figure 3.

So far, our analysis showed that spike-triggered stimulus ensembles covered subspaces of the stimulus space that were specific for particular combinations of kinematic parameters. Virtually all cells showed complex spike-triggered distributions that could be described as separated or partially confluent lobes (indicating different combinations of spike-triggered kinematic variables). However, it turned out that a subset of the observed lobes could be trivially explained by the combination of precise spike patterns (bursts) and stimulus correlation (predictability). This is demonstrated using an SA cell (same as in Figure 3) that showed prominent spike doublets at ~2 ms inter-spike intervals (Figure 6A). We suspected that such doublets which were generated well within the correlation time of our low pass filtered stimulus (~10 ms) led to the partially confluent double-lobe visible at negative velocities in the spike-triggered ensemble (Figure 6B). To reveal the kinematic stimulus variables coded by spike patterns, we first had to identify significant patterns and then show that these patterns were of neuronal origin, independent of stimulus correlation. The latter was realized by calculating the corrected AC of the spike train which eliminated the correlations due to the stimulus from the raw ACs. Significant patterns were identified by peaks that exceeded a PI calculated by a bootstrapping procedure (for both steps see materials and methods). In our example, the corrected AC displayed a prominent peak at ~2 ms time lag, exceeding the PI, and thus, indicated significant doublet firing (Figure 6C). Using the significant lags in the correlated AC as a mask to search through the spike train we identified all doublets in the spike train. The time stamp of the first spike in the doublet was selected as the time stamp of the doublet. We then calculated the single spike and doublet-triggered stimulus ensembles and constructed a map in which we indicated for each bin in stimulus space the composition of the patterns that were evoked by it (Figure 6D, red: simple spikes, green: doublets, shades of yellow: singles and doublets at different ratios, black: <5 events). As expected, one of the two confluent main lobes was abolished by this procedure, revealing that it had held the stimulus ensemble triggered by the second spikes within doublets. As a result, single spikes cover the low-intensity regions of the main lobe as well as the separated smaller lobe while doublets cover the extreme ranges of the main lobe.

**Figure 7:**
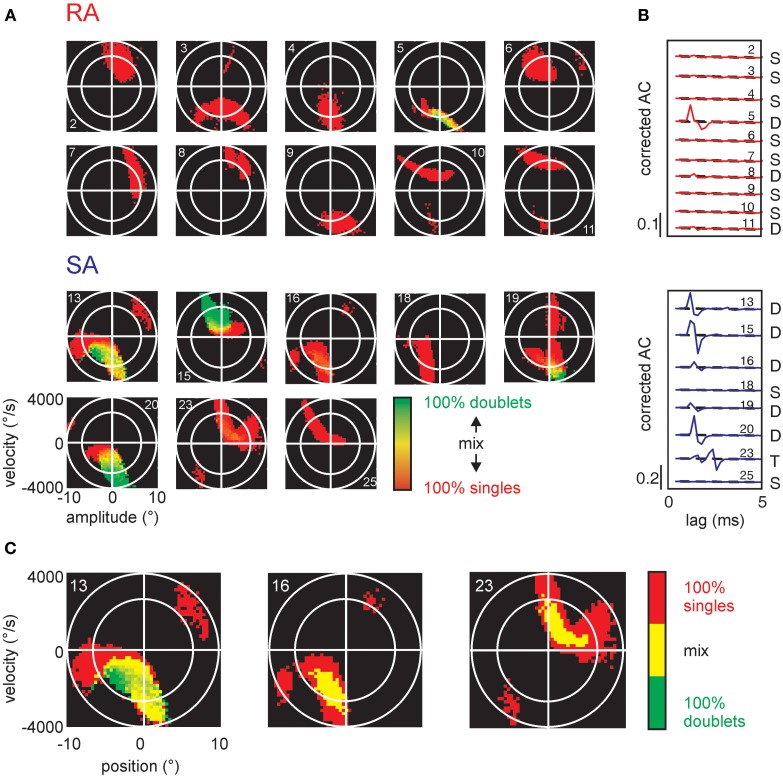
**Stimulus encoding of single spikes and doublets for all cells in the sample. (A)** Conventions as in Figure 6D, all cells in the sample. **(B)** Corrected autocorrelograms of all cells in the sample. Ordinate scales correlation coefficient (*r*). **(C)** The SA cells (numbers 13, 16, and 23) are re-plotted in a different color scheme to visualize the location of doublets and intermixed regions. Here, we only use three colors: pure single spike-triggered stimulus variables are colored red, pure doublet coding is shown in green, and stimulus-triggered by a mix of singles and doublets is colored yellow.

Significant patterns identified in the total sample of cells were all doublets and in one case triplets (cell #23) at precise inter-spike intervals of 1-2 ms (Table 1). The pattern-triggered stimulus ensembles (Figure 7) demonstrate two important characteristics. First, in a large subset of cells (5 of 8 SAs and 4 of 10 RAs), single spikes code for two separate *preferred stimuli* (i.e., for two well separated lobes in stimulus space). Second, doublets (and occasionally triplets) tend to be restricted to one of the lobes and code for extreme stimulus features (i.e., typically higher velocities). Three SA cells (#13, #16, and #23), however, did not fully comply with the second rule. These cells generated doublets with stimuli well in the center of the stimulus space. As doublet coding was mixed in with large numbers of single-spike coding this feature was not revealed by the color plots in Figure 7A. We therefore plot them in a different way (Figure 7B), simply using just three colors indicating pure single spike and doublet coding (i.e., spike-triggered ensembles obtained exclusively with one of the patterns) as well as stimulus regions that are coded by both patterns. Clearly, in these three cases, doublets were generated by stimuli at velocities much lower than the extremes encoded by the cells. In summary, the notion that multi-spike patterns code for stimulus extremes is valid in most but not all cells. On the other hand, single spike and doublet coding is mixed in these deviant cases—not supporting the hypothesis that different patterns code for different preferred stimuli.

**Table 1:** **Numbers of spike patterns as identified with corrected autocorrelograms for each cell (cf. Figure 7)**.

**Cell type**	**Cell no**.	**Singles**	**Doublets**	**Triplets**	**Singles isolated lobe**
RA	02	6947	0	0	0
	03	9833	0	0	141
	04	3684	0	0	0
	05	3541	2229	0	0
	06	4269	0	0	2836
	07	3473	0	0	119
	08	2296	0	0	0
	09	2756	0	0	0
	10	9305	0	0	2697
	11	5163	40	0	193
SA	13	12600	8888	0	616
	15	7188	14403	0	13
	16	11755	1173	0	122
	18	11458	0	0	0
	19	21275	2207	0	0
	20	8292	9278	0	52
	23	19133	1535	22	275
	25	18161	0	0	0

### Explaining information rate—instantaneous coding of kinematic variables

**Figure 8:**
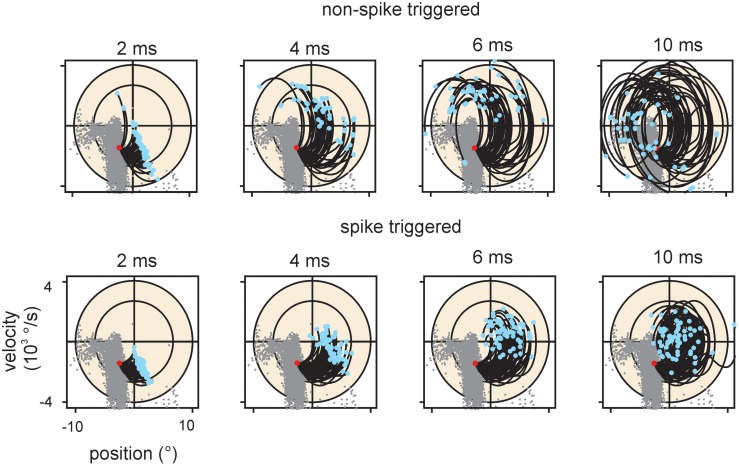
**Encoding of stimulus trajectory**. The graphs plot trajectories ending at a location in stimulus space within the main lobe of cell #18. Only the 2D projection spanning position and velocity is shown for clarity. Conventions for axes and ellipsoid in spike triggered ensembles as in previous figures. Trajectory start is marked by blue dots; trajectories end at the red dots. For reference the spike triggered instantaneous stimuli are depicted as gray dots in all graphs. Top row: Trajectories that did not evoke a spike. Bottom row: Trajectories that evoked a spike. All dots (gray, red, and blue) account for the optimal delay of the cell (cf. Figure 3). The duration of the trajectories is indicated above the graphs. Spike triggered trajectories (bottom) are more confined in the stimulus space as compared to the non-spike triggered ones (top), suggesting that the spikes carry trajectory information.

The information carried about instantaneous kinematic variables was measured using the KLD analysis at optimal delays between trajectory and spike as depicted above (Figure 3). The aim was to assess to what extent coding of position (p), velocity (v), and acceleration (a) can account for the information rate estimated by the DM. Position and acceleration in a white noise stimulus are negatively correlated, and thus information carried by these two variables is at least partially redundant. In order to separate their contributions, we calculated information carried about all possible combinations of kinematic parameters. Position alone and acceleration alone led to inconsistent and non-causal results with optimal latencies between 0 and +10 ms (i.e., on the trajectory that followed the spike) and were not further analyzed. We therefore started to analyze information about velocity alone (v), then its combination with position (pv), and lastly the triple combination of position, velocity and acceleration (pva). Information about the combination velocity and acceleration (va) was calculated as well, but consistently yielded lower information rates than pv. All information rates were tested for significance by a bootstrapping procedure (1000 resampling steps) based on identical analysis but using spike trains in which the inter-spike intervals have been permutated. From the bootstrapped distributions, 5–95 percentile PIs were assessed. All information rates about v, pv, va, and pva lay far outside the respective PIs, indicating significant encoding. Information increased considerably when comparing encoding of v against pv, but rather moderately when comparing pv to pva (although these comparisons reached significance in the sign rank test, see Table 2). Compared to the information rate obtained with DM1.1, the instantaneous encoding is on average 22, 40, 43% (v, pv, pva) for SA and 31, 43, 51% for RA. We conclude that instantaneous coding of kinematic variables does not fully explain information transfer in trigeminal afferents. Examining short strips of stimulus trajectories (10 ms length) that elicited a spike vs. trajectories that did not, revealed that the first are more confined in stimulus space compared to the latter, suggesting that primary afferent spikes transfer information about trajectory within a short time span before the spike occurrence (Figure 8). In summary, we present evidence that (i) the multiple lobed structure, (ii) the trajectory within larger time intervals, and (iii) spike patterns may play a role in primary afferent coding. In the next section, these possibilities will be incorporated into a probabilistic model with the aim to explain primary afferent information rate better than that achieved by instantaneous coding alone.

**Table 2:** **Instantaneous information rates about instantaneous kinematic parameters of whisker deflection**.

	**SA (*n* = 8)**		**RA (*n* = 10)**	
	**Median**	**[5–95%]**	**Median**	**[5–95%]**
v	76.48	54.31–140.05	42.26	16.73–80.25
pv	151.47	110.88–193.01	58.99	22.61–117.65
pva	167.36	116.12–204	66.121	30.46–140.63

v, velocity; p, position; a, acceleration. All numbers are bits/s. Pairwise comparisons between v-pv and vp-pva are significant at p < 0.01 (signrank test) for SA and RA cells.

### Explaining information rate with the spike-triggered mixture model (STM)

**Figure 9:**
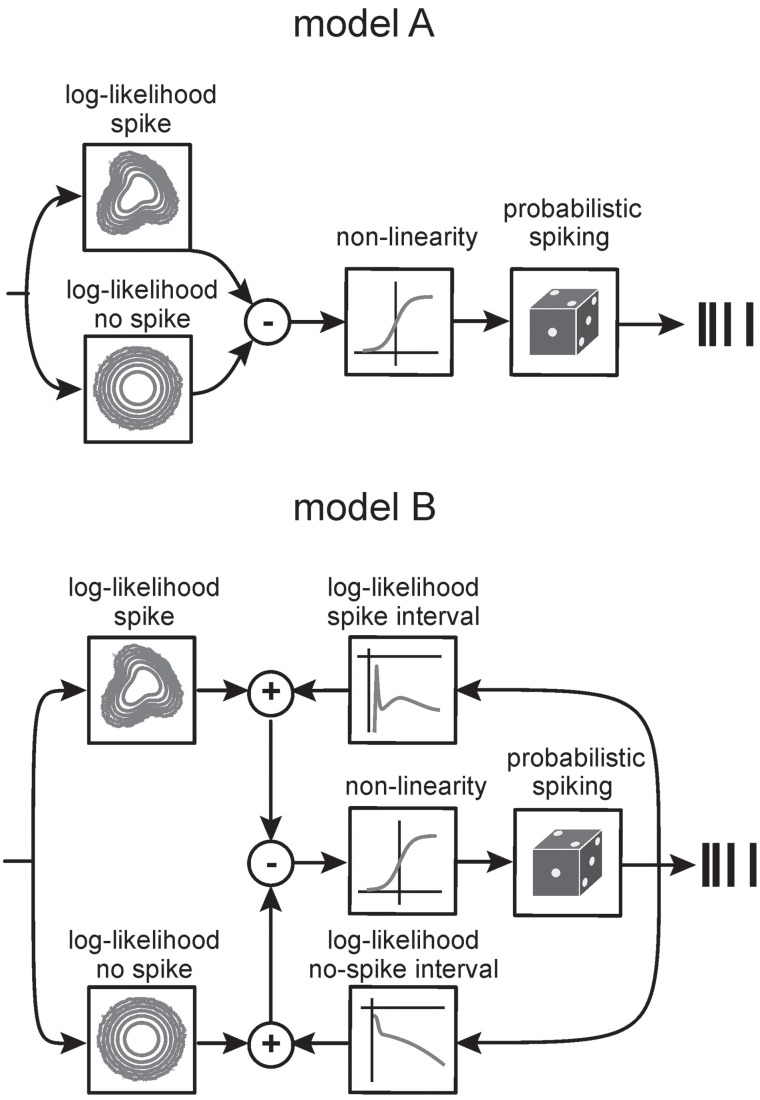
**Generative encoding model based on mixtures of Gaussians (STM model). Model A**. Non-linear features derived from the fits of spike-triggered and non-spike-triggered distributions by multiple Gaussians (boxes on the left) are linearly combined and the response passed through a sigmoid point non-linearity. The result determines the firing rate of the neuron. **Model B**. An extension of the model to incorporates spike-time dependencies. Log-densities of observing certain inter-spike intervals are plotted in the top right. The box on the lower right shows the log-density for observing intervals between a bin with no spike and a spike. The four log-likelihood terms are combined in a principled manner to give the firing rate of the neuron. For details of the model, see Theis et al. (2013).

**Figure 10:**
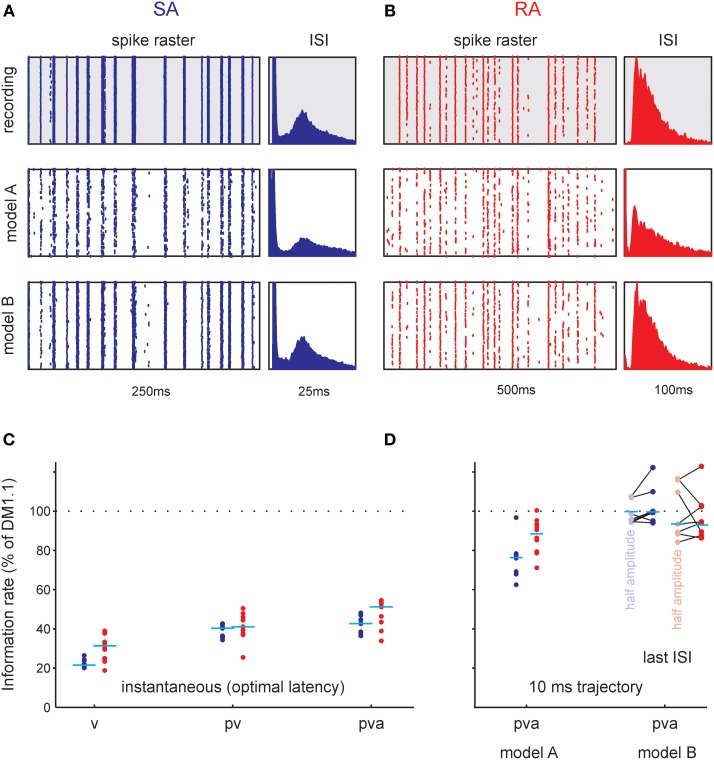
**Functional analysis of total information rate using the STM model. (A,B)** Raster plots (left) and inter-spike interval distributions (ISI, right) as recorded (gray background), and generated by two probabilistic models (white background, cf. Figure 9). **(A)** Typical SA cell. Each row corresponds to one trial with the frozen white noise stimulus. Model A ignores the spike intervals. It reproduces the raster fairly well but introduces too many small intervals. Model B takes the spike intervals into account and clearly reproduces the cells' spike train better. Furthermore, it captures the refractory period and the doublet spiking of the cells better, as can be seen from the inter-spike-interval histograms (right). **(B)** Same as **(A)** but for a typical RA cell. **(C,D)**. Comparison of information rate with DM1.1. Data from all cells; horizontal lines depict medians. **(C)** Information rate for instantaneous encoding of kinematic variables (p, position; v, velocity; a, acceleration). The information rate conveyed about combinations v, pv, and pva (that gave consistent and causally plausible delays of spikes following the stimulus and spikes) as normalized to the rate estimated by DM1.1 are shown. **(D)** Information rate relative to DM1.1 estimated using the STM model. Model A uses a 10 ms trajectory as input but ignores the spike history. The information rate is higher than in the instantaneous case but clearly lower than DM1.1. Accounting in addition for the last spike interval (Model B) results in information rates that are comparable with the ones obtained using DM1.1. The results plotted in lighter color were obtained from spike trains sampled during presentation of stimuli that were identical but roughly of half amplitude (3 standard deviations 5.1° instead of 10°, lines connect data points sampled from the same neurons).

As multiple preferred stimuli will likely be insufficiently captured by kernel-based methods like stimulus reconstruction (Bialek et al., 1991) and inherently linear models such as the commonly used linear non-linear Poisson model, we used an alternative approach that captures the lobed structure by fitting a mixture of Gaussians to the stimulus distributions (STM, Figure 9, **Model A**, *cf.* Theis et al., 2013). As described in the method section, the STM model can be used to quantify information rates, and can capture complex dependencies on the stimulus. In addition, the STM model is able to accommodate a spike-history dependent term which allows it to model bursting (Equations 7, 8, **Model B** in Figure 9). Figure 10 compares the output generated by the neuron model with and without a spike-history-dependent term (top row: recorded cell, middle row: Model A, bottom row: Model B) for a representative SA and RA cell (panels A and B, respectively). Raster displays and inter-spike-interval histograms of Model B clearly match both observed neurons' spike trains better. Figure 10C plots the information rate achieved with instantaneous coding at the neurons' optimal delays, expressed as the percentage of information achieved with the DM. This is compared to the estimated information rate after accounting for the trajectory in a pre-spike interval of 10 ms but ignoring spike history (model A) in Figure 10D. The median information rate estimated with model A was 289.73 bit/s (2.82 bit/spike) for SA cells (maximum: 416.20 bit/s, 4.49 bit/spike) and 116.14 bit/s (4.80 bit/spike) for RA cells (maximum: 244.55 bit/s, 6.41 bit/spike). To fully explain the information rate calculated from DM1.1 we added information about spike history. It turned out that adding information about the last spike was enough to explain all information calculated by DM1.1. The median estimated information rate was 373.77 bit/s (3.70 bit/spike) for SA cells (maximum: 541.38 bit/s, 5.08 bit/spike) and 142.64 bit/s (5.79 bit/spike) for RA cells (maximum: 302.32 bit/s, 7.65 bit/spike) which compares well with the results obtained with DM1.1. In order to find out whether the fraction of DM1.1 calculated information explained by STM is dependent on the stimulus amplitude, we repeated the calculation using data obtained with filtered white noise stimuli of roughly half amplitude (3 standard deviation of 5.1° instead of 10°) in a subset of cells. As expected the absolute information flow was reduced with the smaller stimulus (DM1.1: RA: 67.32 bit/s, SA: 346.02 bit/s; STM: RA: 67.68 bit/s; SA: 353.73 bit/s), but the ratio of information estimates obtained with DM1.1 and STM stayed largely the same (Figure 10D). In summary, the information rate obtained with instantaneous encoding is about 50% of that measured with DM1.1. Adding the 10 ms pre-spike trajectory, the model reaches 80–90%. Finally, around 100% of DM1.1 information rate is captured when additionally incorporating the dependency of spike intervals in bursts.

## Discussion

### Explaining information rate

Our quantitative analysis revealed information rates reaching up to 529 bit/s (7 bit/spike)—amongst the highest reported so far with a frequency limit of 100 Hz (minimum resolution of 10 ms). Previous evidence suggests that the temporal precision of spiking in TG reaches down to 1 ms (Jones et al., 2004a). The literature entails a multitude of studies measuring information rates and precision, sometimes employing the DM, but most commonly using stimulus reconstruction methods (Bialek et al., 1991). To our knowledge, the highest published rate so far is that of photoreceptors of diurnal bees and mechanoreceptors of cockroaches, both of which reach about 500 bit/s, matching the information rate found here (French and Torkkeli, 1998; Frederiksen et al., 2008). Other sensory receptors and afferents have been reported to transfer less but still above 180 bit/s, amongst them frog and grass hopper auditory fibers (Rieke et al., 1995; Machens et al., 2001), cricket mechanosensory receptors (Roddey and Jacobs, 1996), chordodontal proprioceptive afferents of the shore crab (Dicaprio et al., 2007), and P-type electroreceptor afferents in electric fish (Wessel et al., 1996). Transfer ranges below 100 bit/s were observed in turtle vestibular canal afferents, and electroreceptor afferents of paddlefish (Neiman et al., 2011; Rowe and Neiman, 2012). Many of these cells show encoding limits lower than 10 ms. Information rates measured in central neurons of different animals ranging from insects up to monkeys are much smaller than the ones typically found for sensory receptors (see for overview: Borst and Theunissen, 1999; fly H1 and monkey MT, (Strong et al., 1998); retinal ganglion cells (Koch et al., 2004; Passaglia and Troy, 2004); LGN neurons, (Sincich et al., 2009); V1 simple cells, (Reich et al., 2001). From these published data it seems reasonable to assume that peripheral receptors in general are optimized to convey maximal sensory information at fast rates. We demonstrate here that the information rate of whisker-related primary afferents is within the top ranges of this list and the temporal resolution of less than 10 ms is on par with that of other systems. This supports and quantifies the notion that coding in whisker-related primary afferents and subsequent ascending pathways is fast and precise (Jones et al., 2004a; Petersen et al., 2008; Stüttgen and Schwarz, 2010). In this study, however, we wanted to go beyond the rather abstract quantification of information rates. We were particularly interested in explaining the constituents of the information rate, i.e., determining which specific physical parameters are encoded by the tactile system, and to what extent.

The common existence of spike patterns together with coding for multiple preferred stimuli (and thus a non-monotonic relationship between stimulus and response) are captured well by modeling the spike-triggered stimulus ensemble using a mixture of Gaussians (Theis et al., 2013). The mechanistic insights gained with this novel method go far beyond the abstract information rate offered by DM, and also greatly exceed that of the current gold standard in modeling neuronal encoding, the linear-non-linear Poisson (LNP) model (*cf.* Theis et al., 2013). Kernels of LNP models fitted to responses of thalamic neurons to filtered white noise whisker deflection suggest contributions of position, velocity, and acceleration (Petersen et al., 2008). However, the details of their influence remain vague due to the inevitable temporal stimulus correlation introduced by mechanical constraints (i.e., the need to low-pass filter the stimulus. Appendix 1). We wish to emphasize that the multiple lobes we demonstrate here correspond directly to modes within the spike-triggered distribution itself, a case not captured by standard LNP analysis (Schwartz et al., 2006). With LNP analysis, the observation of a multi-modal link function (e.g., a quadratic non-linearity as is typically found for complex cells in the visual cortex, Schwartz et al., 2006) does not necessarily imply a multi-modal response subspace. The reason is that the link function is related to the ratio between spike-triggered and prior distribution, which allows e.g., the bi-modal quadratic function to be generated from two unimodal distributions with different variances. The KLD approach allowed us to conclude that instantaneous encoding captured only about 50% of the total information (using DM1.1 as a benchmark). Velocity and position are the variables principally encoded by primary afferents, with acceleration contributing only marginally. The STM model indicated that another 40% of the DM1.1 information rate are contributed by 10 ms pre-spike trajectory segments. And finally, coding with doublets could easily be accommodated: The last 10% are captured by the dependence of the spike probability on the proceeding spike interval quantifying the contribution of information conveyed by bursting.

### Consequences for whisker-related sensation

Our findings of obliquely oriented spike-triggered lobes and multiple preferred stimuli clearly rejects the notion of pure positional (SA) and velocity (RA) encoders. This view has been questioned earlier because of the fact that positional coding (i.e., the sustained response to step-like stimuli) has been found to be largely lost on the ascending pathway toward the barrel cortex (Shipley, 1974; Simons and Carvell, 1989; Lee et al., 1994a,b; Veinante and Deschênes, 1999; Hartings et al., 2003; Minnery and Simons, 2003; Hemelt and Keller, 2007; Stüttgen and Schwarz, 2008). Furthermore, encoded features obtained from linear filter based methods like LNP models (Petersen et al., 2008) or stimulus reconstruction techniques (Jones et al., 2004a) often display signatures compatible with both positional as well as velocity encoding. Finally, behavioral work revealed that psychophysical channels based on SA and RA cells convey specific combinations of step amplitude and velocity (Stüttgen et al., 2006). Despite these coding properties, the notion that the two cell classes provide the basis for the perceptual coverage of a vast stimulus range as suggested from responses to step-like stimuli (Shoykhet et al., 2000; Stüttgen et al., 2006) was confirmed, as SA and RA encode different and complementary subspaces along the velocity axis (Figure 4C).

The finding that both cell classes generate specific types of bursts (mostly doublets) adding to the information of single spikes has important consequences for the readout of primary afferent information by the trigeminal nuclei (TN)—the next station on the ascending pathway. We wish to emphasize, however, that the majority of features encoded by different spike patterns are contiguous in the stimulus space, indicating that bursts are not used as qualitatively different encoding symbols. In other words, doublets typically do not code for an entirely different aspect of the stimulus, but simply for higher velocities than single spikes. In all cells showing doublets, there is a transition zone in which the stimulus evokes a mix of singles and doublets. At present, detailed characteristics of the EPSPs evoked by primary afferents in recipient TN neurons are unknown. Therefore, to decide whether and how doublets and triplets are conveyed up the ascending tactile pathway, more information about temporal overlap of EPSPs, short-term plasticity, and TN network interactions are needed. Our finding that single spikes are generated in response to multiple preferred stimuli (the separate lobes in the spike-triggered stimulus ensembles) posits a real challenge for any readout mechanism. The resulting ambiguity of coding at the single cell level must likely be eliminated by information from the population responses of many primary afferents innervating the same follicle. The cellular mechanism responsible for the generation of the multi-lobed structure is unknown. Two possibilities are that either the biomechanics of the end organ attached to the follicle's glassy membrane (Ebara et al., 2002) are complex and give rise to the separation of preferred stimuli. Detailed realistic mechanical models of the end organs in rat vibrissae follicles are missing and are needed for future work to gain insight into this question. The second possibility is that one primary afferent innervates multiple separate end organs (differing either in location or type). For this purpose, knowledge about structure-function relationships of primary afferents and innervated end-organs are needed, best obtained by using intracellular recording and fillings of TG cells. Finally, we wish to emphasize that neuronal coding is adaptive (Fraser et al., 2006; Maravall et al., 2007). Adaptive coding implies that predictions from response fields obtained with one stimulus are not necessarily met by responses obtained with another. Appendix 2 shows that this applies also to trigeminal afferents.

In summary, the primary afferents provide the whisker-related tactile pathway with an enormous amount of stimulus information. This information is typically encoded in a non-linear, adaptive fashion and occurs occasionally in a non-monotonic, i.e., multiple-preferred-stimulus fashion. It will be important to find out how stations on the ascending pathway read out this information.

### Conflict of interest statement

The authors declare that the research was conducted in the absence of any commercial or financial relationships that could be construed as a potential conflict of interest.

## Figures and Tables

**Figure A1:**
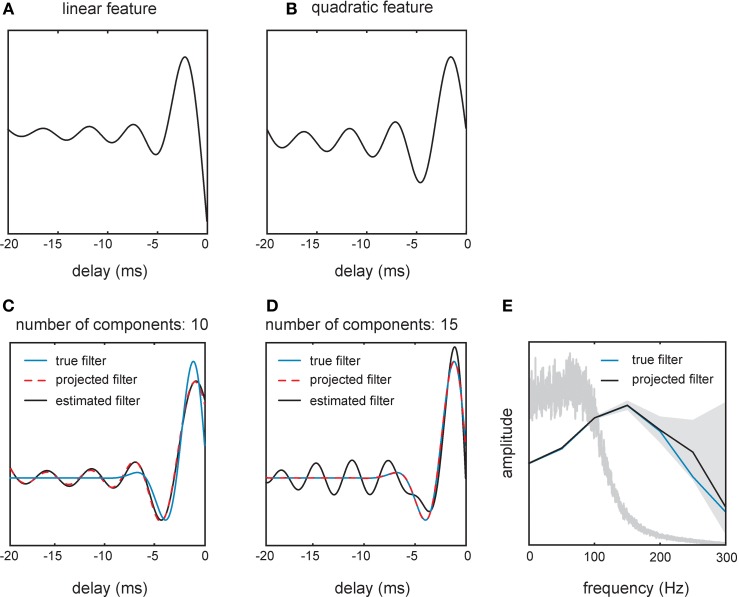
**Filter shapes estimated from spikes generated by one real and one artificial cell in response to a 100 Hz filtered white noise stimulus. (A,B)** show filters obtained by fitting an STM to the responses of one SA cell. **(C)** The stimulus filter of a toy neuron (blue line) was estimated (black line) from the model's responses to the same stimulus as used in the experiments with real cells. Since we only used 10 principal component vectors to represent the filter, even a perfect estimate (yellow dashed line) would not have been able to recover the true filter. **(D)** Adding higher-frequency components allows us to represent the true filter but also increases the error in the estimated filter. **(E)** Amplitude spectra of the true filter (blue line) and the estimated filter (black solid line) of the toy neuron. The shaded region indicates a 90% confidence interval revealing high uncertainty in the high-frequency components of the estimated filter. The spectrum of the stimulus is shown as a gray line for reference (cf. Figure 2C).

**Figure A2:**
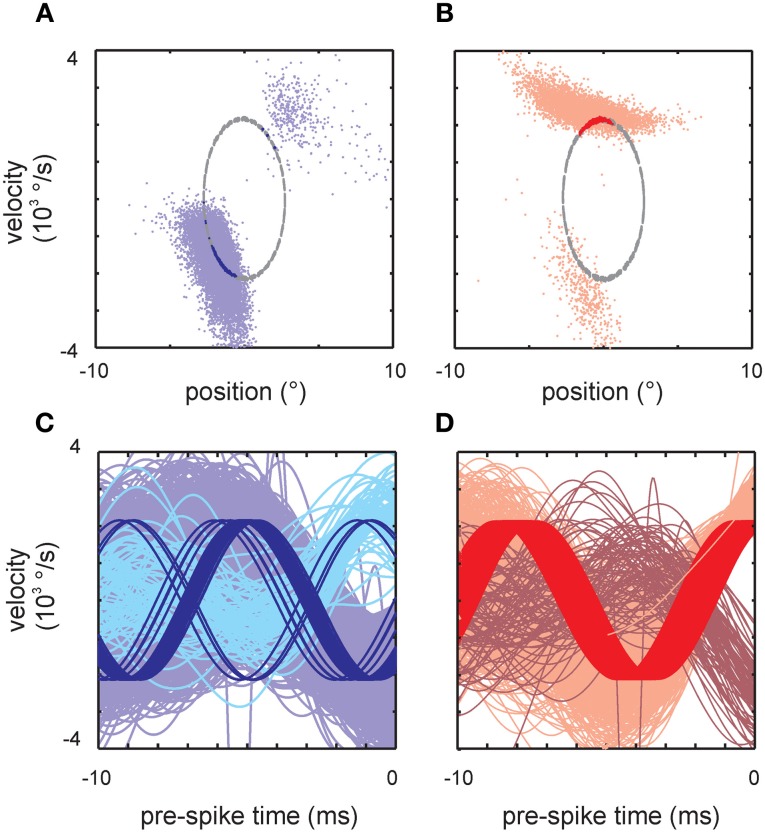
**Non-predictability of responses to simple stimuli from complex stimuli. (A,B)** Responses to sine waves (on the ellipsoid track through the 2D kinematic space) and band-pass limited white noise of two example cells. **(A)** Primary afferent (SA, cell 16) that responded to the sine wave when entering both lobes as characterized by the broad-band stimulus. Such predictability was only seen in 2 cells **(B)** Another cell (RA, cell 11) did not respond when the sine wave traversed the second lobe (seen in 6 cells). **(C,D)** Same data but now plotted to reveal the velocity trajectory of broad-band and sine wave stimuli within the 10 ms interval preceding the spike. **(C)** Same cell as (**A)**. The broad-band trajectories landing in the two lobes are depicted in light and medium blue color. The spike triggered sine wave trajectories are plotted in dark blue. **(D)** Same as **(C)**, but for the cell shown in **(B)** (light and medium red: broad-band trajectories; dark red: sine wave trajectories). Here, in contrast to **(C)**, sine waves landing in the lower lobe never elicit spikes.

## Appendix A–filter estimation

It is common practice to interpret the shape of estimated model filters as e.g., integrators (i.e., responsive to position) or differentiators (i.e., responsive to velocity) (cf. Petersen et al., 2008; Estebanez et al., 2012). We did not analyze model parameters in the present study because the quality of estimated filter shapes is highly dependent on our ability to estimate the full range of encoded frequencies from the applied stimulus. Ideally, the stimulus should contain all frequencies encoded by the studied neuron, in which case the estimation of all aspects of the filters is possible. However, with tactile stimuli this optimal setting is currently unachievable because of mechanical limitations of actuators (e.g., inertia, resonance frequencies, etc.). White noise approaches to study the whisker system to date, all have to use noise stimuli limited to frequencies below 200 Hz, which doubtlessly under-represent the range of encoded frequencies. Judging from phase locking to sinusoidal stimuli, encoded frequencies may well reach up to 1 kHz (Deschenes et al., 2003).

Here we demonstrate the effect of a deficient stimulus on the estimated filter shapes. Filters have traditionally been estimated in the context of linear-non-linear models (e.g., Schwartz et al., 2006). Similarly, fitting an STM model as done in this article involves the estimation of filters. According to Theis et al. (2013), the input to the sigmoid of an STM, *f*(*x*, τ) (Equation 8), can be re-parameterized as

(10) $$f\text{​}\left(x,\tau \right)=\log\sum_{k}\text{exp}\text{​}\left(\sum_{m}\beta_{km}{\left(u_{m}^{⊤}x\right)}^{2}+w_{k}^{⊤}x+\alpha_{k}\right)+h_{\tau },$$

where *u*_*m*_ and *w*_*k*_ are vectors of the size of the stimulus and β_km_, *a*_k_ and *h*_τ_ are scalars. Unlike linear models, the STM model has several filters which can be grouped into linear features *w*_k_ and quadratic features *u*_m_. Figures A1A,B show examples of estimated filters represented using only the first 10 principle components of the stimulus. What strikes the eye are the strong oscillations, which, as we will show below, are most likely due to the 10 principal components' inability to represent the high frequency components of the optimal filter. That is, the oscillations are in fact ringing artifacts.

To illustrate the problem of ringing we used a “toy neuron” realized by a generalized linear model (GLM) with a sigmoid non-linearity and a Bernoulli output distribution. Responses of the toy neuron were generated using the same band-limited white noise stimulus as used to drive the real neurons. The advantage here is that the optimal filter of the toy neuron is known and represents ground truth (Figures A1C–E). We estimated the filter of the toy neuron by fitting a randomly initialized GLM to the responses via maximum likelihood learning. After estimating and reconstructing the filter using the first 10 principal components, we observed the same oscillatory behavior of the filter as with the STM. Importantly, the projection of the true filter using the same 10 principle components gave the same result (Figure A1C). This demonstrates that the oscillations are in fact ringing artifacts and not due to errors in the estimate. Therefore, the learned filters approximate a distorted version of the true filter as seen when reducing it to 10 dimensions (i.e., ignoring its high frequency components). Using more principle components (*n* = 15) allowed us to fully represent the true filter (Figure A1D). However, as shown in the representation of the filter in the frequency domain (Figure A1E), estimates of the high frequency components of the filter were noisy, resulting in even higher oscillations in the estimated filter (Figure A1D). We conclude from these considerations that filter estimation given the limitations of tactile stimulus presentation is imprecise and prone to ringing artifacts.

### Methodological details

Confidence intervals on the filter amplitude spectra (gray field in Figure A1E) were obtained as follows. Under certain conditions and as the amount of training data increases, maximum likelihood estimates are known to be asymptotically Gaussian distributed with a covariance which corresponds to the inverse of the Fisher information matrix of the optimal parameter values. We computed the Fisher information matrix as

(11) $$I(\theta )=\sum_{n}\nabla_{\theta }\log p(s_{n}|x_{n},\tau_{n},\theta )\cdot \nabla_{\theta }\log p\text{​}{\left(s_{n}|x_{n},\tau_{n},\theta \right)}^{⊤},$$

where the index *n* runs over data points and the gradient was evaluated at the maximum likelihood estimate of the parameters θ. To obtain 90% confidence intervals for the amplitude spectrum, we sampled 10^4^ filters from the Gaussian distribution over filters with the maximum likelihood estimate as the mean and the inverse Fisher information matrix as the covariance and computed their amplitude spectra. 5 and 95% percentiles of the amplitude for each frequency were taken as the 90% confidence interval.

## Appendix B–adaptive coding

Here we aim to demonstrate that response properties obtained with band-pass limited white noise do not necessarily predict responses to simple stimuli classically used to portray neuronal coding (and vice versa). For instance, bi-lobed responses found with the classical LNP method (i.e., with quadratic non-linearities) have been observed to lead to corresponding phase doubling using sine wave stimulation in barrel cortex (Estebanez et al., 2012). We found that the lobed structure revealed by STM in the primary afferents in the present study does not necessarily predict spike generation with sine wave stimulation (and vice versa). Figures A2A,B shows the responses of two primary afferents (cells 10 and 16) to the band-limited white noise and to the sine wave stimuli used for the classification in SA and RA (cf. Figure 1). Across our sample of 18 cells 6 did not respond to all lobes that the sine wave stimulus traversed (as the cell in Figure A2B), 2 responded to all lobes the sine wave entered (as in Figure A2A), 2 responded outside the lobes, and in 8 cells the sine wave track entered only one lobe and generated spikes. Therefore, in 8 out of 18 cells, we found spike generation in response to sine waves not expected from response fields obtained with white noise stimulation. We next checked if the failure to evoke spikes could be explained by the fact that the sine waves used did not match the encoded 10 ms trajectories (cf. Figures 8, 10D). This was not the case as the employed sine wave quite well matched the encoded trajectories, but still did not evoke spikes (Figures A2C,D). In conclusion, we wish to emphasize that the absence or presence of phase doubling observable with sine wave stimulation does not allow to make predictions about a primary afferent's lobed response. The opposite is also not true: the presence of multi-lobed responses with complex stimuli does not predict phase doubling with sine waves.

## References

[B1] Bialek W. Rieke F. De Ruyter Van Stefenick R. R. Warland D. (1991). Reading a neural code. Science 252, 1854–1857 10.1126/science.2063199 2063199

[B1a] Bishop C. M. (2006). Pattern Recognition and Machine Learning. New York, NY: Springer

[B2] Borst A. Theunissen F. E. (1999). Information theory and neural coding. Nat. Neurosci. 2, 947–957 10.1038/14731 10526332

[B3] De Ruyter Van Steveninck R. R. Lewen G. D. Strong S. P. Koberle R. Bialek W. (1997). Reproducibility and variability in neural spike trains. Science 275, 1805–1808 10.1126/science.275.5307.1805 9065407

[B4] Deschenes M. Timofeeva E. Lavallee P. (2003). The relay of high-frequency sensory signals in the Whisker-to-barreloid pathway. J. Neurosci. 23, 6778–6787 12890771 10.1523/JNEUROSCI.23-17-06778.2003 PMC6740730

[B5] Dicaprio R. A. Billimoria C. P. Ludwar B. (2007). Information rate and spike-timing precision of proprioceptive afferents. J. Neurophysiol. 98, 1706–1717 10.1152/jn.00176.2007 17634343

[B6] Ebara S. Kumamoto K. Matsuura T. Mazurkiewicz J. E. Rice F. L. (2002). Similarities and differences in the innervation of mystacial vibrissal follicle-sinus complexes in the rat and cat: a confocal microscopic study. J. Comp. Neurol. 449, 103–119 10.1002/cne.10277 12115682

[B7] Estebanez L. El Boustani S. Destexhe A. Shulz D. E. (2012). Correlated input reveals coexisting coding schemes in a sensory cortex. Nat. Neurosci. 15, 1691–1699 10.1038/nn.3258 23160042

[B8] Fraser G. Hartings J. A. Simons D. J. (2006). Adaptation of trigeminal ganglion cells to periodic whisker deflections. Somatosens. Mot. Res. 23, 111–118 10.1080/08990220600906589 17178546

[B9] Frederiksen R. Wcislo W. T. Warrant E. J. (2008). Visual reliability and information rate in the retina of a nocturnal bee. Curr. Biol. 18, 349–353 10.1016/j.cub.2008.01.057 18328705

[B10] French A. S. Torkkeli P. H. (1998). Information transmission at 500 bits/s by action potentials in a mechanosensory neuron of the cockroach. Neurosci. Lett. 243, 113–116 10.1016/S0304-3940(98)00110-4 9535126

[B11] Gibson J. M. Welker W. I. (1983a). Quantitative studies of stimulus coding in first-order vibrissa afferents of rats. 1. Receptive field properties and threshold distributions. Somatosens. Res. 1, 51–67 10.3109/07367228309144540 6679913

[B12] Gibson J. M. Welker W. I. (1983b). Quantitative studies of stimulus coding in first-order vibrissa afferents of rats. 2. Adaptation and coding of stimulus parameters. Somatosens. Res. 1, 95–117 10.3109/07367228309144543 6679920

[B13] Hartings J. A. Temereanca S. Simons D. J. (2003). Processing of periodic whisker deflections by neurons in the ventroposterior medial and thalamic reticular nuclei. J. Neurophysiol. 90, 3087–3094 10.1152/jn.00469.2003 14615426

[B14] Hemelt M. E. Keller A. (2007). Superior sensation: superior colliculus participation in rat vibrissa system. BMC Neurosci. 8:12 10.1186/1471-2202-8-12 17266753 PMC1796887

[B15] Jones L. M. Depireux D. A. Simons D. J. Keller A. (2004a). Robust temporal coding in the trigeminal system. Science 304, 1986–1989 10.1126/science.1097779 15218153 PMC1557422

[B16] Jones L. M. Lee S. Trageser J. C. Simons D. J. Keller A. (2004b). Precise temporal responses in whisker trigeminal neurons. J. Neurophysiol. 92, 665–668 10.1152/jn.00031.2004 14999053 PMC2800049

[B17] Koch K. McLean J. Berry M. Sterling P. Balasubramanian V. Freed M. A. (2004). Efficiency of information transmission by retinal ganglion cells. Curr. Biol. 14, 1523–1530 10.1016/j.cub.2004.08.060 15341738

[B18] Lee S. M. Friedberg M. H. Ebner F. F. (1994a). The role of GABA-mediated inhibition in the rat ventral posterior medial thalamus. I. Assessment of receptive field changes following thalamic reticular nucleus lesions. J. Neurophysiol. 71, 1702–1715 8064343 10.1152/jn.1994.71.5.1702

[B19] Lee S. M. Friedberg M. H. Ebner F. F. (1994b). The role of GABA-mediated inhibition in the rat ventral posterior medial thalamus. II. Differential effects of GABAA and GABAB receptor antagonists on responses of VPM neurons. J. Neurophysiol. 71, 1716–1726 8064344 10.1152/jn.1994.71.5.1716

[B20] Machens C. K. Stemmler M. B. Prinz P. Krahe R. Ronacher B. Herz A. V. (2001). Representation of acoustic communication signals by insect auditory receptor neurons. J. Neurosci. 21, 3215–3227 11312306 10.1523/JNEUROSCI.21-09-03215.2001 PMC6762569

[B21] Maravall M. Petersen R. S. Fairhall A. L. Arabzadeh E. Diamond M. E. (2007). Shifts in coding properties and maintenance of information transmission during adaptation in barrel cortex. PLoS Biol. 5:e19 10.1371/journal.pbio.0050019 17253902 PMC1779810

[B22] Minnery B. S. Simons D. J. (2003). Response properties of whisker-associated trigeminothalamic neurons in rat nucleus principalis. J. Neurophysiol. 89, 40–56 10.1152/jn.00272.2002 12522158

[B23] Neiman A. B. Russell D. F. Rowe M. H. (2011). Identifying temporal codes in spontaneously active sensory neurons. PLoS ONE 6:e27380 10.1371/journal.pone.0027380 22087303 PMC3210806

[B24] Nocedal J. Wright S. J. (1999). Numerical Optimization. New York, NY: Springer 10.1007/b98874

[B25] Passaglia C. L. Troy J. B. (2004). Information transmission rates of cat retinal ganglion cells. J. Neurophysiol. 91, 1217–1229 10.1152/jn.00796.2003 14602836 PMC5130245

[B26] Petersen R. S. Brambilla M. Bale M. R. Alenda A. Panzeri S. Montemurro M. A. (2008). Diverse and temporally precise kinetic feature selectivity in the VPm thalamic nucleus. Neuron 60, 890–903 10.1016/j.neuron.2008.09.041 19081382

[B27] Reich D. S. Mechler F. Victor J. D. (2001). Formal and attribute-specific information in primary visual cortex. J. Neurophysiol. 85, 305–318 11152730 10.1152/jn.2001.85.1.305

[B28] Rieke F. Bodnar D. A. Bialek W. (1995). Naturalistic stimuli increase the rate and efficiency of information transmission by primary auditory afferents. Proc. Biol. Sci. 262, 259–265 10.1098/rspb.1995.0204 8587884

[B29] Roddey J. C. Jacobs G. A. (1996). Information theoretic analysis of dynamical encoding by filiform mechanoreceptors in the cricket cercal system. J. Neurophysiol. 75, 1365–1376 8727383 10.1152/jn.1996.75.4.1365

[B30] Rowe M. H. Neiman A. B. (2012). Information analysis of posterior canal afferents in the turtle, Trachemys scripta elegans. Brain Res. 1434, 226–242 10.1016/j.brainres.2011.08.016 21890114 PMC3233658

[B31] Schwartz O. Pillow J. W. Rust N. C. Simoncelli E. P. (2006). Spike-triggered neural characterization. J. Vis. 6, 484–507 10.1167/6.4.13 16889482

[B32] Shipley M. T. (1974). Response characteristics of single units in the rat's trigeminal nuclei to vibrissa displacements. J. Neurophysiol. 37, 73–90 4359792 10.1152/jn.1974.37.1.73

[B33] Shoykhet M. Doherty D. Simons D. J. (2000). Coding of deflection velocity and amplitude by whisker primary afferent neurons: implications for higher level processing. Somatosens. Mot. Res. 17, 171–180 10.1080/08990220050020580 10895887

[B34] Simons D. J. Carvell G. E. (1989). Thalamocortical response transformation in the rat vibrissa/barrel system. J. Neurophysiol. 61, 311–330 2918357 10.1152/jn.1989.61.2.311

[B35] Sincich L. C. Horton J. C. Sharpee T. O. (2009). Preserving information in neural transmission. J. Neurosci. 29, 6207–6216 10.1523/JNEUROSCI.3701-08.2009 19439598 PMC2761742

[B36] Strong S. P. De Ruyter Van Steveninck R. R. Bialek W. Koberle R. (1998). On the application of information theory to neural spike trains. Pac. Symp. Biocomput. 3, 621–632 9697217

[B37] Stüttgen M. C. Schwarz C. (2008). Psychophysical and neurometric detection performance under stimulus uncertainty. Nat. Neurosci. 11, 1091–1099 10.1038/nn.2162 19160508

[B38] Stüttgen M. C. Schwarz C. (2010). Integration of vibrotactile signals for whisker-related perception in rats is governed by short time constants: comparison of neurometric and psychometric detection performance. J. Neurosci. 30, 2060–2069 10.1523/JNEUROSCI.3943-09.2010 20147534 PMC6634023

[B39] Stüttgen M. C. Kullmann S. Schwarz C. (2008). Responses of rat trigeminal ganglion neurons to longitudinal whisker stimulation. J. Neurophysiol. 100, 1879–1884 10.1152/jn.90511.2008 18684907

[B40] Stüttgen M. C. Rüter J. Schwarz C. (2006). Two psychophysical channels of Whisker deflection in rats align with two neuronal classes of primary afferents. J. Neurosci. 26, 7933–7941 10.1523/JNEUROSCI.1864-06.2006 16870738 PMC6674210

[B40a] Theis L. Chagas A. M. Arnstein D. Schwarz C. Bethge M. (2013). Beyond GLMs: A Generative Mixture Modeling Approach to Neural System Identification. PLoS Comput. Biol. 9:e1003356 10.1371/journal.pcbi.1003356 24278006 PMC3836720

[B41] Veinante P. Deschênes M. (1999). Single- and multi-whisker channels in the ascending projections from the principal trigeminal nucleus in the Rat. J. Neurosci. 19, 5085–5095 10366641 10.1523/JNEUROSCI.19-12-05085.1999 PMC6782641

[B42] Wessel R. Koch C. Gabbiani F. (1996). Coding of time-varying electric field amplitude modulations in a wave-type electric fish. J. Neurophysiol. 75, 2280–2293 8793741 10.1152/jn.1996.75.6.2280

[B43] Zhang H. (2004). The optimality of naive Bayes, in Proceedings of the 17th International FLAIRS Conference (FLAIRS2004) (Miami Beach, FL: AAAI Press).

